# Validation of a model-based virtual trials method for tight glycemic control in intensive care

**DOI:** 10.1186/1475-925X-9-84

**Published:** 2010-12-14

**Authors:** J Geoffrey Chase, Fatanah Suhaimi, Sophie Penning, Jean-Charles Preiser, Aaron J Le Compte, Jessica Lin, Christopher G Pretty, Geoffrey M Shaw, Katherine T Moorhead, Thomas Desaive

**Affiliations:** 1 Dept of Mechanical Eng, Centre for Bio-Engineering, University of Canterbury, Christchurch, Private Bag 4800, 8054, New Zealand; 2 Cardiovascular Research Centre, Institute de Physique, Universite de Liege, Institute of Physics, Allée du 6 Août, 17 (Bât B5), B4000 Liege, Liege, Belgium; 3 Dept of Intensive Care, Erasme University Hospital, 808 route de Lennik, B1070 Brussels, Belgium; 4 University of Otago Christchurch, School of Medicine, Christchurch, 8054, New Zealand; 5 Dept of Intensive Care, Christchurch Hospital, Christchurch, 8054, New Zealand

## Abstract

**Background:**

*In-silico *virtual patients and trials offer significant advantages in cost, time and safety for designing effective tight glycemic control (TGC) protocols. However, no such method has fully validated the independence of virtual patients (or resulting clinical trial predictions) from the data used to create them. This study uses matched cohorts from a TGC clinical trial to validate virtual patients and in-silico virtual trial models and methods.

**Methods:**

Data from a 211 patient subset of the Glucontrol trial in Liege, Belgium. Glucontrol-A (N = 142) targeted 4.4-6.1 mmol/L and Glucontrol-B (N = 69) targeted 7.8-10.0 mmol/L. Cohorts were matched by APACHE II score, initial BG, age, weight, BMI and sex (p > 0.25). Virtual patients are created by fitting a clinically validated model to clinical data, yielding time varying insulin sensitivity profiles (SI(t)) that drives *in-silico *patients.

Model fit and intra-patient (forward) prediction errors are used to validate individual *in-silico *virtual patients. Self-validation (tests A protocol on Group-A virtual patients; and B protocol on B virtual patients) and cross-validation (tests A protocol on Group-B virtual patients; and B protocol on A virtual patients) are used in comparison to clinical data to assess ability to predict clinical trial results.

**Results:**

Model fit errors were small (<0.25%) for all patients, indicating model fitness. Median forward prediction errors were: 4.3, 2.8 and 3.5% for Group-A, Group-B and Overall (A+B), indicating individual virtual patients were accurate representations of real patients. SI and its variability were similar between cohorts indicating they were metabolically similar.

Self and cross validation results were within 1-10% of the clinical data for both Group-A and Group-B. Self-validation indicated clinically insignificant errors due to model and/or clinical compliance. Cross-validation clearly showed that virtual patients enabled by identified patient-specific SI(t) profiles can accurately predict the performance of independent and different TGC protocols.

**Conclusions:**

This study fully validates these virtual patients and *in silico *virtual trial methods, and clearly shows they can accurately simulate, in advance, the clinical results of a TGC protocol, enabling rapid in silico protocol design and optimization. These outcomes provide the first rigorous validation of a virtual *in-silico *patient and virtual trials methodology.

## Introduction

Stress-induced hyperglycemia and high levels of insulin resistance are prevalent in critical care [1-4]. Increased counter-regulatory hormone secretion stimulates endogenous glucose production and increases insulin resistance [3,4], elevating equilibrium glucose levels and reducing the amount of glucose the body can utilize with a given amount of insulin. Hyperglycemia worsens outcomes, increasing the risk of severe infection, myocardial infarction, and critical illness polyneuropathy and multiple organ failure.

The occurrence of hyperglycemia, particularly severe hyperglycemia, is associated with increased morbidity and mortality [2]. Glycemic variability and poor control are independently associated with increased mortality [5-7]. Van den Berghe et al [8,9] showed that tight glucose control (TGC) reduced intensive care unit (ICU) patient mortality up to 45% using a target of 6.1 mmol/L. Other studies with similar or slightly higher targets have successfully reduced mortality [10,11]. Hence, despite some difficulty repeating these results [12], the data indicate that a control algorithm that safely provides TGC to reduce hyperglycemia and glycemic variability can reduce mortality and cost [13,14].

In this study, "virtual trials" are performed using a clinically validated model [15-17] of the glucose-insulin system. Insulin sensitivity, *S_I_*, is used as the critical marker of a patient's metabolic state and is assumed independent of the insulin and nutrition inputs. Virtual trials can be used to simulate a TGC protocol using a *S_I_(t) *profile identified hourly from clinical data and different insulin and nutrition inputs. Virtual trials enable the rapid testing of new TGC intervention protocols, as well as analysis with respect to glycemic control protocol performance, safety from hypoglycaemia, clinical burden, and the ability to handle dynamic changes in patient metabolic state [15,18]. They are thus a means of safely optimising protocols prior to clinical implementation.

Virtual patient trials have been used in design of TGC protocols [16,19-21]. Others have developed them for evaluating type 1 diabetes treatments [22,23] and in critical care [24], but none have been specifically validated in comparison to clinical trial or patient-specific outcomes. Specific to this study, the clinical results of SPRINT [11] showed very close agreement to expected results from simulation [16,21]. However, SPRINT was implemented in the Christchurch Hospital ICU, and all the clinical data, models and virtual trial methods used to design it were from the same unit so it is not an independent ICU in that sense.

Thus, the performance of virtual trials on separate matched cohorts has not yet been evaluated. In addition, the assumption of the independence of a virtual patient's insulin sensitivity *S_I_(t) *profile from the insulin and nutrition inputs used to identify it from clinical blood glucose (BG) data has never been validated. This study tests these assumptions using clinically matched (virtual) cohorts based on clinical data from an independent ICU, who were treated with two different glycemic control protocols in a randomised trial. The independence of the ICU ensures a cohort who may be different in treatment, insulin sensitivity or other factors [25] from patients in the Christchurch ICU whose data underlie the development of the models and methods [16,19-21] validated in this study. Hence, there is no link between the patients used in this study and the development of the models and methods being tested here. Hence, these clinically matched cohorts allow this assumption of independence to be tested, as well as the assessment of model errors in this virtual trial approach.

## Methods

### Glucontrol Protocol and Patient Cohorts

The Glucontrol trial [26] randomised patients into two groups: Group A and Group B. Group A received intensive insulin therapy and Group B received conventional insulin therapy, with target ranges of 4.4-6.1 mmol/L and 7.8-10 mmol/L, respectively. Insulin was administered as a continuous intravenous (IV) infusion. Hourly blood glucose (BG) measurements were recorded when the glycemic level was not within the target range. Otherwise, 2-hourly measurements were taken in the case of limited variation of glycemia, defined as less than a 50% change from the previous glycaemia in 2-hour range. Finally, 4-hourly measurements were taken when the glycemic level was less than 50% of the highest glycemia of the four last hours. If other BG measurements were taken, they were not recorded and did not result in changes to the insulin infusion rate. The protocol specified insulin infusion rates are shown in Table 1 for the intensive protocol used on Group A, and Table 2 for the conventional protocol used on Group B. Nutritional input was left to local and/or clinician standards, and was not explicitly considered in the Glucontrol TGC protocols.

**Table 1:** Glucontrol Group A protocol (intensive). The starting insulin infusion rate is in the top portion and the maintenance insulin infusion rates and increments are in the bottom portion as labelled. All values converted to mmol/L from mg/dL in 27.

STARTING INSULIN INFUSION RATE SCALE	
**Glycemia**	**Starting insulin infusion rate**

<6.1 mmol/L	On hold

6.1 - 7.8 mmol/L	1 U/h

7.8 - 10.0 mmol/L	2 U/h

>10.0 mmol/L	4 U/h

**MAINTENANCE INFUSION RATE CHANGES**	

**Glycemia**	**Incremental insulin infusion rate**

>16.7 mmol/L	+3 U/h

10.0 - 16.7 mmol/L	+2 U/h

7.8 - 10.0 mmol/L	+1 U/h

6.1 - 7.8 mmol/L	+0.5 U/h

4.4 - 6.1 mmol/L	+0 U/h (target range)

2.2 - 4.4 mmol/L	Stop insulin, Hourly measurement of glycemia until >80 mg/dl

<2.2 mmol/L	Stop insulin, 10 gr glucose IVD, Call physician immediately, Hourly measurement of glycemia until >80 mg/dl

**Table 2:** Glucontrol Group B protocol (conventional). (a) Starting insulin infusion rate. (b) Maintenance insulin infusion rates and increments. All values converted to mmol/L from mg/dL in 27.

STARTING INSULIN INFUSION RATE SCALE	
**Glycemia**	**Starting insulin infusion rate**

<10.0 mmol/L	On hold

10.0 - 13.9 mmol/L	1 U/h

13.9 - 16.7 mmol/L	2 U/h

>16.7 mmol/L	4 U/h

**MAINTENANCE INFUSION RATE CHANGES**	

**Glycemia**	**Incremental insulin infusion rate**

>16.7 mmol/L	+3 U/h

13.9 - 16.7 mmol/L	+2 U/h

10.0 - 13.9 mmol/L	+1 U/h

7.8 - 10.0 mmol/L	+0 U/h (target range)

4.4 - 7.8 mmol/L	Decrease 50% rate insulin

2.2 - 4.4 mmol/L	Stop insulin, Hourly measurement of glycemia until >80 mg/dl

<2.2 mmol/L	Stop insulin, 10 gr glucose IVD, Call physician immediately, Hourly measurement of glycemia until >80 mg/dl

**Figure 1:**
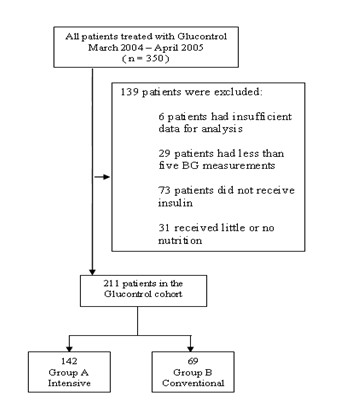
**Cohort selection for Glucontrol A (Intensive) and B (Conventional) insulin therapy groups, resulting in 211 total Glucontrol patients being retained from the original 350**. Note that each arm of the trial (A and B) each had 175 patients, so that 33 were removed from Group A and 106 from Group B.

In this study, data was used from 350 patients (175 in each arm) treated using the Glucontrol protocol at CHU de Liege, Belgium, between March 2004 and April 2005. Thus, the Glucontrol data used in this study is from only one centre out of the full study [26]. The selection criteria for patients used in this analysis to generate virtual patients with sufficient data density [15,16,27] are shown in Figure 1. Patients were eliminated from the analysis if they received no insulin for their entire stay (per protocol), had less than 5 BG measurements or received little or no (recorded) carbohydrate administration (in any form) for more than 48 hours of their stay.

Clinical details of the resulting cohorts are in Table 3 totalling 29,777 hours and 7,391 BG measurements. Patients in Group A were slightly older than Group B. However, there were no significant differences in sex, weight, BMI, severity of illness as measured by APACHE II score or initial BG level. Group B received less insulin and more carbohydrate, in alignment with its higher glycemic target.

**Table 3:** Glucontrol Group A and B comparison. P-values are computed using chi-squared and Mann-Whitney tests. values are median [IQR] as appropriate.

Cohort	A	B	*P *value
**Baseline Variables**			

Number patients	142	69	
Male percent (%)	64.8	56.5	0.25
Age	71 [61 - 80]	69 [53 - 77]	0.035
Weight	72 [62 - 85]	75 [68 - 81]	0.38
BMI	25.4 [22.6 - 29.3]	26.0 [23.2 - 29.3]	0.46
APACHE II	17 [14 - 22]	17 [14 - 21]	0.76
Initial BG (mmol/L)	6.6 [5.6 - 8.6]	6.6 [5.7 - 9.4]	0.58

**Glucose Control**			

Total hours	16, 831	12, 946	
BG measurements	4, 571	2, 820	
BG (mmol/L)	6.3 [5.3 - 7.6]	8.2 [6.9 - 9.4]	
Insulin rate (U/h)	1.5 [0.5 - 3.0]	0.7 [0.0 - 1.7]	
Carbohydrate admin (all sources) (mmol/min)	0.30 [0.00 - 0.90]	0.60 [0.10 - 1.00]	

### Glucose-Insulin System Model

The analysis of patient-specific insulin sensitivity uses a glucose insulin system model that has been clinically validated in several clinical TGC studies [17,19,28-30]:

(1) $$\dot{G}=-p_{G}G-S_{I}G\frac{Q}{1+\alpha_{G}Q}+\frac{P(t)+EGP_{\max}-CNS}{V_{G}(t)}$$

(2) $$\dot{Q}=-kQ+kI$$

(3) $$\dot{I}=-\frac{nI}{1+\alpha_{I}I}+\frac{u_{ex}(t)}{V_{I}}+e^{-k_{I}U_{ex}(t)}I_{B}$$

where: *G(t) *is total plasma glucose, *I(t) *is plasma insulin, and *Q(t) *is the effect of previously infused insulin being utilized over time. *EGP_max _*is the theoretical maximum endogenous glucose production (EGP), which is suppressed with increasing glucose concentrations. This suppression, independent of non-insulin mediated glucose uptake by the central nervous system (*CNS*) is captured by the term *p_G_*. In contrast, patient-specific insulin mediated glucose removal is captured with insulin sensitivity, *S_I_*, which is identified (hourly) from clinical data as a time-dependent variable that reflects evolving patient condition [15,18,27,31]. Exogenous inputs are glucose appearance *P(t) *from the carbohydrate content of nutrition infusions via a two compartment model [19], and intravenous insulin administration *u_ex_(t)*. The remaining parameters are physiologically defined population constants for transport rates (*n*, *k*), saturation parameters (*α_G_*, *α_I_*), endogenous insulin secretion (*I_B_*, *k_I_*) or volumes (*V_G_*, *V_I_*) that have been validated over several studies.

The essential parameter that drives the observed patient-specific glycemic response to insulin and nutrition inputs is insulin sensitivity, *S_I_*. It is identified by fitting the model to BG measurements, and insulin and carbohydrate administration inputs, from retrospective clinical data for each protocol [27,32]. The resulting insulin sensitivity profile, *S_I_(t)*, identifies a unique value every hour and the resulting profile thus varies hourly. This model-based, insulin sensitivity metric and identification method have been validated in TGC clinical trials in adults and neonates [17,19,28-30] and *S_I _*has also shown good correlation to gold standard euglycemic clamp data [33,34].

Sensitivity analysis has shown this approach can capture the clinically observed dynamics and variation. Median BG data fit and 1-2 hour forward prediction errors are within 2-10% compared to measurement errors of 7-12%. Hence, this model and methods have been used to analyse, develop and/or implement new protocols [16-18,21,25,28-30].

### Insulin Sensitivity Dynamics

The dynamics of insulin sensitivity variability can be compared between cohorts. Kernel density modeling provides a smooth, physiologically realistic description of the hour-to-hour changes in insulin sensitivity, *S_I _*[15,18,35]. Confidence intervals for these stochastic models of hourly variation can be visually compared to indicate whether cohorts exhibit similar insulin sensitivity, and thus metabolic, variability [25], as a further, clinically relevant and important comparator of TGC cohorts.

### Virtual Trials

**Figure 2:**
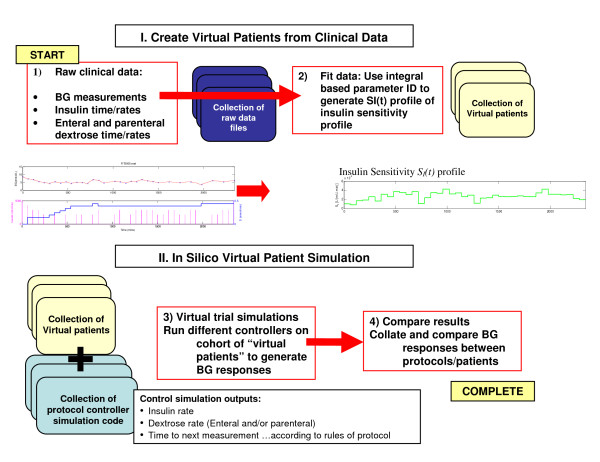
**Virtual patient development and in silico simulation method**. (I) Clinical data are used for fitting insulin sensitivity profiles to create 'virtual patients'. (II) These virtual patients can be used for simulating different protocols.

The "virtual trial" method is used to simulate a trial using patient specific data. The insulin sensitivity profile *S_I_(t) *identified from clinical data captures a patient-specific time varying glycemic response to the given insulin and nutrition inputs. This *S_I_(t) *profile can then be used to simulate the blood glucose response to other combinations of insulin and dextrose inputs specified by a modified TGC protocol to obtain new in silico glycemic responses [16]. Hence, an expected blood glucose profile can be generated for each patient to simulate patient-specific glycemic responses to a specific protocol. Thus, virtual trials can be used to analyse, in silico, the effect of different TGC protocols on patient-specific glycemic performance. Figure 2 shows this overall process.

The critical assumption is that the identified *S_I_(t) *profiles are (largely) independent of the clinical data used to derive them. In this analysis, 2 groups of virtual patients are created from randomised clinical trial data. Groups A and B are defined by whether they were clinically treated with either the Glucontrol A (intensive) or Glucontrol B (conventional) TGC protocols. Using matched cohorts treated differently allows this assumption to be tested. The hypothesis is thus defined: if *S_I_*(t) is independent, similar control results would be achieved in the cross-validation.

### Validation Analysis

This study performs three major forms of validation using virtual trials. These three tests provide both per-patient and cohort-wide validation of this in silico approach.

#### 1) Model Fit and Prediction Error

Model fit and prediction errors are used to show the ability of the model to fit the data and predict the expected patient state. Using the identified *S_I _*profiles, the simulated BG measurements were compared to clinical BG data. This fit error quantifies the ability of the model to capture the observed dynamics.

Prediction results are generated by holding insulin sensitivity, *S_I _*constant for an upcoming hour, and simulating the BG one hour into the future using the recorded clinical insulin and dextrose inputs. This BG prediction is compared to clinically recorded BG or a linear estimate between 2-hourly measurements. Prediction errors assess the ability of the model and identified parameters to predict intervention outcomes and is highly relevant for validating models used in model-based TGC [16,36].

Prediction errors are thus an assessment of the model's ability to make accurate patient-specific prediction. Given low fit errors it thus also assesses whether the identified and given model parameters are accurate. In this study, prediction error serves to validate the model identification method [27] and approach [16,21] used to create virtual patients and virtual trials.

#### 2) Self-Validation

Self validation tests the ability of the in silico virtual patient modelling method to reproduce the clinical data from which a virtual cohort was derived. For the self validation on Glucontrol A, the Glucontrol A protocol defined in Table 1 is simulated on Group A virtual patients, and these virtual trial results are compared to the clinical data from Group A. This step was repeated for self validation on Glucontrol B.

Differences between clinical and virtual trial results can be ascribed to model errors, and/or lack of perfect compliance in the clinical study versus the perfect compliance and timing in silico. Hence, two self-validation virtual trials were simulated on each group considering: a) the actual measurement timing used in the clinical trials (actual measurement) and b) measurement timing from the protocol (per protocol). Comparing actual and per-protocol measurement timing allows one to assess one aspect of compliance error.

For self validation of actual measurement, the timing used in the virtual trial strictly follows the measurement timing in the clinical trials where the controller selects the proper intervention in response to the BG values at the exact time correspond to its clinical time. In contrast, per protocol self validation follows exactly the Glucontrol A and B protocols defined in Tables 1 and 2 regardless of the measurement timing they had clinically. The controller will still select the intervention according to the current BG values. However, because the Glucontrol protocols modify insulin by increments to a prior infusion rate in Tables 1 and 2, different measurement timing could significantly change dosing, which would thus indicate the impact of compliance to measurement timing.

#### 3) Cross-Validation

Cross validation uses the matched A and B cohorts to determine the ability of the modelling method to reproduce the clinical data on a matched, but independent, cohort. Thus, Protocol A is simulated on virtual patients derived from Group B clinical data, with results compared to clinical data from Glucontrol Group A. Similarly, Protocol B is tested on virtual patients from Group A and the results are compared to Group B clinical data.

In theory, if patients were perfectly matched in all ways, the in silico and clinical data would also match if the in silico virtual trials method were exact. Differences using large matched cohorts can thus be largely ascribed to how well the assumption holds that these virtual patient *S_I_(t) *profiles are independent of the clinical insulin and nutrition inputs used to derive them. If cross validation results match the clinical results well, for clinically matched cohorts, then this assumption can be considered valid. Hence, this validation tests the underlying assumption of this virtual trial method.

**Figure 3:**
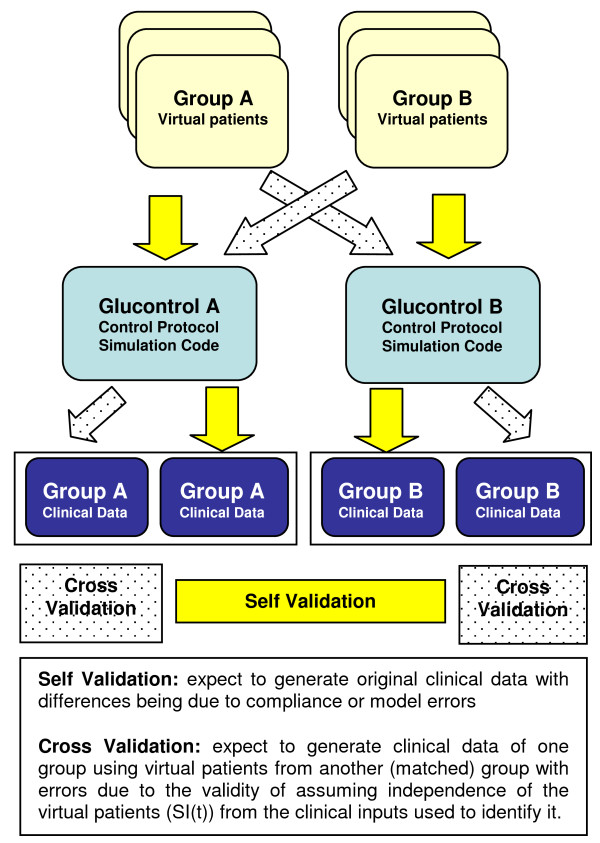
**Virtual trial validation method**. These profiles are then used to re-simulate the Glucontrol A and Glucontrol B protocols for comparison to the appropriate clinical results.

Figure 3 shows the self-validation and cross-validation processes schematically.

## Results

### Metabolic Variability

**Figure 4:**
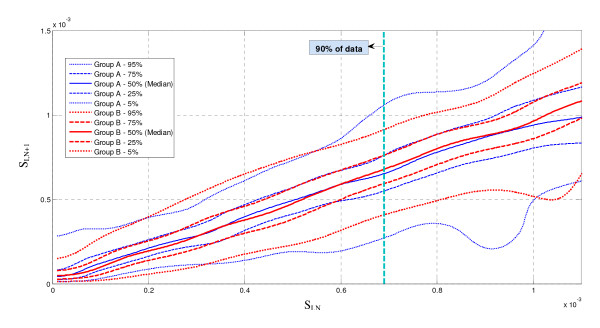
**Hourly *S*I variation and stochastic probability distribution for Group A and Group B**. Stochastic models are generated for data where the BG measurement interval was 1-2 hourly [22].

Figure 4 shows the 5^th ^- 95^th ^percentile range, IQR and median probability bounds for stochastic models for Group A and Group B. The distributions indicate the hour to hour intra-patient metabolic variability in *S_I _*is very similar across the majority of the *S_I _*range, particularly for the middle 50% (IQR). In particular, 89%-93% of the data for both groups was in the range 0.01 × 10^-3 ^≤ *S_I _*≤ 0.8 × 10^-3^, which is where there was greatest agreement between the groups. Above this range sparse data had an effect, particularly on the 5% and 95% bounds. Hence, the clinically matched cohorts of Table 3 are also similar in metabolic variability, which is significant evidence of similar metabolic response and variability in response to insulin across patients and cohorts that is important in this analysis.

### Fit, Prediction Validation

**Figure 5:**
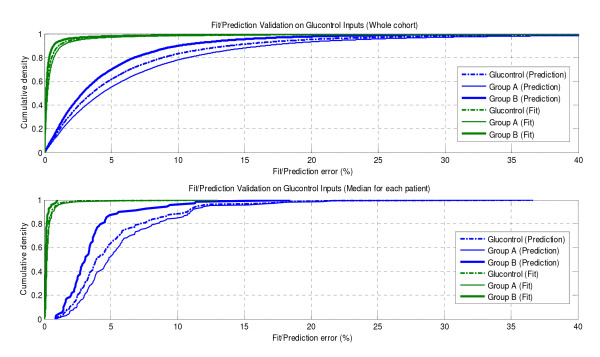
**Plot of prediction errors for Group A, Group B and entire Glucontrol cohort**. Model fit errors essentially overlaid for all 3 cohort groupings.

Figure 5 shows the model fit and prediction errors for the entire Glucontrol cohort (A + B), and separated into Group A and Group B. Results are shown on a cohort and a per-patient basis. Model fit error was consistent across all three groups analysed, with median fit error <0.25% in all cases. Group B has the lowest prediction error among these three distributions. The Glucontrol (A + B) cohort prediction error median value was 3.5%, whereas Group A and Group B were 4.3% and 2.8%, respectively. All these median errors are below typical sensor errors of 7-12%.

Prediction errors are a function of hour-to-hour patient variability and ability of the model to accurately capture insulin and glucose dynamics. Figure 4 indicates variability in insulin sensitivity is similar (within 2% for all ΔS_I_) for the cohorts. Thus, the model prediction errors for Group A and B have 80% or more of their results ≤10% measurement error, despite significant differences in clinical insulin usage in Table 3. This performance across different cohorts is similar in a clinical sense where relatively smaller errors of 10-12%, or differences in error of 2-5% are not clinically significant in outcome. However, it should be noted that they are not statistically the same. Similar results are seen for the median patient fit and prediction errors in the lower panel of Figure 5.

### Self & Cross Validation

**Figure 6:**
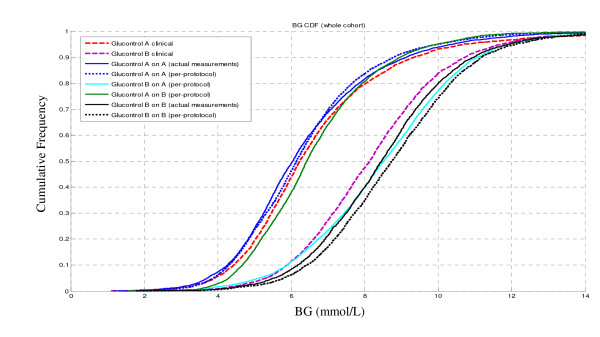
**CDF of blood glucose levels of clinical Glucontrol data versus virtual trials on a cohort basis**. The A and B cohort sets of (3) curves are labeled.

Figure 6 shows the CDF of measured blood glucose on a cohort basis, comparing clinical data from Glucontrol A and B to:

1. Self validation: Per protocol and actual measurement virtual trial results for Glucontrol A and B on the Group A and Group B virtual patients.

2. Cross validation: Virtual trial results for the Glucontrol A protocol on Glucontrol B virtual patients, and the Glucontrol B protocol on virtual Group A patients.

The breakdown of distributions shows a clear separation between the Glucontrol A and Glucontrol B protocols, as expected from the clinical results, and equally for all combinations of simulations.

The four distributions for the Glucontrol A protocol show particularly close agreement. The Glucontrol A clinical median cohort BG value of 6.2 mmol/L agrees well with the 6.0 mmol/L and 6.2 mmol/L medians for the self validation trials using actual and per-protocol BG measurement timing respectively. The cross-validation median BG of 6.5 mmol/L is also in close agreement with the clinical result.

The four BG distributions for the Glucontrol B protocol show a slightly greater spread in results, particularly below the Group B target of 8 mmol/L. However, the median cohort clinical BG value of 8.1 still agrees well with the medians of 8.5 and 8.7 mmol/L for Glucontrol B self validation with actual and per-protocol measurement frequency, respectively. It also agrees well with the cross-validation median result of 8.5 mmol/L. The cross validation result lies between the clinical data and self validation result indicating it is within the model and/or compliance error compared to the clinical data.

Finally, the wider error in the Glucontrol B protocol results may be due to the relatively low median insulin doses of <1 U/hr (Table 3). Hence, model error grows due to the fixed endogenous insulin rate assumed for *I_B _*in this situation and similarly fixed value assumed for *EGP_max_*. Finally, as with the Group A results, the self and cross validation agreement is within measurement error and clinically insignificant over the CDF.

**Figure 7:**
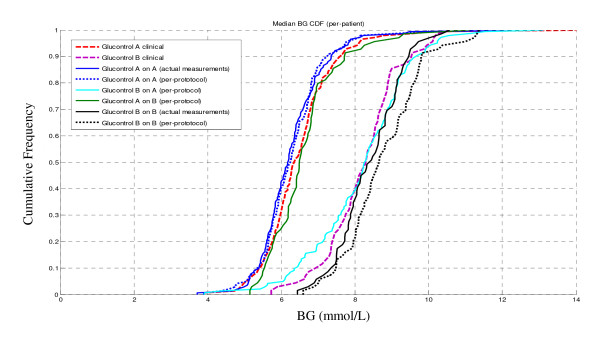
**CDF of median blood glucose measured of Glucontrol clinical data versus virtual trial on a per patient basis**. The A and B cohort sets of (3) curves are labeled.

Figure 7 shows the same results for the CDF of the median patient blood glucose levels across all patients in each group. This "per-patient" comparison has the same whole-cohort trend in Figure 6. Interestingly, and as with the cohort results, the largest gap is between self validation and clinical data for Glucontrol B.

Overall, the gap between the self validation using actual measurement timing and clinical data indicates the possibility of compliance error. In contrast, the difference between self validation simulations using exact protocol-specified timing and the clinical data shows one possible indication of model error. However, it may also suggest that the conventional, lower intensity Group B protocol may not have been followed as strictly with respect to dosing.

Table 4 shows the comparison of clinical trials to the self validation and cross validation on Glucontrol A. Per patient results show a reasonably close agreement between self validation per protocol to the clinical data but the insulin rates are higher given the almost 2× higher measurement rate when using the protocol-specified rules. Using the actual measurement rate, the insulin rates are closer.

**Table 4:** Comparison of per-patient clinical results and virtual trial simulations (self-validation and cross validation) on Glucontrol A. Clinical and actual measurements were taken 52.0% of the potential per-protocol specified times based on in silico glycemic results. Median [IQR] is used where appropriate.

		Self validation		Cross validation
	**Clinical**	**Actual Measurement**	**Per-Protocol**	**Per-Protocol**

No. of patients	142	142	142	69

**Per Patient Results**				

Insulin rate (U/h)	1.4 [0.9 - 2.1]	1.8 [1.1 - 2.9]	2.5 [1.5 - 4.1]	4.5 [2.3 - 6.5]
Glucose rate (g/h)	1.1 [0.5 - 7.6]	1.1 [0.5 - 7.6]	1.1 [0.5 - 7.6]	2.9 [0.7 - 7.4]
BG (mmol/L)	6.4 [5.9 - 6.9]	6.2 [5.7 - 6.8]	6.2 [5.7 - 6.8]	6.5 [6.0 - 6.9]
BG measures	4564	4564	9467	7259
Measurement frequency (measurement/patient/day)	6.52	6.52	13.54	13.48

Differences in measurement rate and insulin dose can be ascribed to non-compliance and due to the design of Glucontrol, where the rate of change of insulin dose is tied to BG measurement frequency. In particular, the clinical and actual measurements were taken 52.0% of the potential per-protocol specified times based on *in silico *glycemic results for Glucontrol A and 63.5% for Glucontrol B in Table 5. Note that Glucontrol B had a higher compliance rate (% of per-protocol measurements) likely due to its higher glycemic target, which allowed 4 hour measurements to start sooner than for Glucontrol A. Thus, it could be construed that Glucontrol A clinical staff were less compliant to a potentially more burdensome protocol in this regard.

**Table 5:** Comparison of per-patient clinical results and virtual trial simulations (self-validation and cross validation) on Glucontrol B. Clinical and actual measurements were taken 63.5% of the potential per-protocol specified times based on glycemic results. Median [IQR] is used where appropriate.

		Self validation		Cross validation
	**Clinical**	**Actual measurement**	**Per protocol**	**Per protocol**

No. of patients	69	69	69	142

**Per Patient Results**				

Insulin rate (U/h)	0.6 [0.3 - 1.2]	0.5 [0.2 - 1.0]	0.6 [0.2 - 1.4]	0.2 [0.0 - 0.8]
Glucose rate (g/h)	2.9 [0.7 - 7.4]	2.9 [0.7 - 7.4]	2.9 [0.7 - 7.4]	1.1 [0.5 - 7.5]
BG (mmol/L)	8.3 [7.6 - 8.8]	8.4 [7.8 - 9.1]	8.7 [8.1 - 9.5]	8.3 [7.4 - 9.1]
BG measures	2820	2820	4448	5772
Measurement frequency (measurement/patient/day)	5.23	5.23	8.23	8.23

For the cross validation, the Glucontrol A protocol required almost 3× higher rates of insulin for Group B, compared to the clinical data. However, this may be a function of the interaction of protocol and measurement frequency where there was a 1.4× difference that results from per protocol versus actual measurement self-validation. That said, the Glucontrol B patients received 2.6× greater carbohydrate input to offset much of this difference in insulin administration. Specifically, the cross validation in Table 4 required 3.2× more insulin to offset 2.6× more carbohydrate administration. Adjusting by 2.5/1.8 the per-protocol versus actual measurement increase in insulin administered yields an estimated 2.4× increase in insulin use to offset this increased carbohydrate administration. Hence, the increased insulin in cross validation in Table 4 is primarily due to the increased carbohydrate administered to Group B.

Comparison of clinical trials with self validation and cross validation on Glucontrol B is summarized in Table 5. Self validation results show close agreement to the clinical result and for cross validation lower Group A insulin requirements are reflected by the lower nutrition given that group and the higher target BG target under the Glucontrol B protocol, which is similar to the difference in insulin in the cross validation in Table 4 but in the reverse direction. Similarly, virtual trials of Glucontrol B per protocol have higher measurement frequency compared to the actual measurement indicating significant non-compliance. Thus, the actual measurement case indicates closer agreement with the insulin given and glycemic outcomes, as with the Glucontrol A results.

## Discussion

This paper focuses on the Glucontrol protocol from one centre (Liege, Belgium; pilot centre). Glucontrol was a multi-centre study stopped early due to a high rate of unintended protocol violations [26]. Hence, some self-validation errors may be the result of poor compliance, as seen in the results of Tables 4 and 5, and thus the different virtual trials can capture that reality. Patient-specific compliance levels ranged from 100% compliance to 20%. However, these values are skewed by length of stay and initial glycemic levels among other possible factors, and thus per-patient statistics are only broadly meaningful.

The clinical data was independent from the Christchurch Hospital ICU data used in prior development and clinical validation of the model employed here. More importantly, there are 2 cohorts matched by severity of illness, weight and sex, which had significantly different glycemic targets and glycemic control therapies. In addition, Figure 4 shows that cohorts appear well matched in their metabolic dynamics and variability which is the critical aspect for this study as it determines the outcome glycemia and variability for a set of given interventions.

One possible limitation is that unequal numbers of virtual patients are created from each cohort (A = 142 of 175 are used; B = 69 of 175), as seen in Figure 1. The reason is that the higher glycemic target of Glucontrol B, and lower compliance, meant that far more patients did not meet the criteria in Figure 1 required to create valid virtual patients due to low data density. However, Table 3 shows that these patients are still matched clinically in the most relevant clinical parameters for survival (APACHE II and initial BG). Additionally, as noted in the results and shown in Figure 4 insulin sensitivity and its variation are similar. As a result, the virtual patients are also equivalent in underlying data quality and clinically important metrics. However, a larger cohort would allow more detailed cross validation on sub-cohorts. Hence, this potential limitation does not appear to skew the results presented or their validity.

Further, despite significant differences between the two protocols, the hour-to-hour intra-patient variation between cohorts is very similar, indicating hour-to-hour changes in insulin sensitivity are patient-specific and protocol-independent. The cohorts can thus be considered interchangeable for the purpose of the cross validation presented. This result also helps independently validate the assumption that this model-based insulin sensitivity is independent of the clinical inputs used to identify it, which is important as this assumption is the basis of these virtual trials.

The model fit errors in Figure 5 are relatively very small and almost overlaid for Group A, B and the entire Glucontrol cohort. The model prediction validation results in Figure 5 can be seen as an estimate of the variability of insulin sensitivity in this cohort, as well as a sign of model fitness. Low 1-hour prediction errors compared to sensor error of 7-12% were found for both groups. For context, this result also suggests the use of model-based targeted BG control will be effective for these cohorts of critically ill patients, as demonstrated previously for Christchurch ICU cohorts upon whom this model was derived and used [16,19,36]. Thus, they also serve as an independent validation of this model using different ICU cohorts. Note that similar, but larger, errors for 2-4 hour predictions have been found for this model [27]. The growth of such error is largely due to the greater chance of significant variation in *S_I _*over longer time periods as the hourly variations in Figure 4 compound. However, it should be noted that in this scenario, 1-hour and 2-hour predictions are clinically relevant.

The distribution of clinically measured BG values shows a very clear difference between Glucontrol protocol A and B, as expected. The virtual trials results are within 5% (median) of the clinical results for both the self validation and cross validations (Figures 6 and 7). Referring to the same figures, the obvious separation between two protocols indicates the inter-protocol differences are, as expected, much larger than any inter-group differences thus supporting the fundamental assumptions behind this virtual trials approach. More importantly, the close correlation of self and cross validation results to clinical data validates the idea that these in silico virtual trial simulations can accurately predict the expected clinical results of a TGC protocol prior to clinical implementation.

The results in Figures 6 and 7 illustrate some variation between clinical data and virtual trials. In particular, Glucontrol A results are closer to the clinical data compared to Glucontrol B. The major difference is that Protocol B uses much less insulin given its higher glycemic target. Therefore, the impact of intrinsic and potentially variable patient-specific dynamics, such as endogenous insulin production (*I_B _*and *k_I_*) and endogenous glucose production (*EGP_max_*), are more pronounced with respect to the far lesser exogenous insulin given to Group B, especially at blood glucose levels below 8.0 mmol/L. As these metrics are unidentifiable and thus, by necessity, assumed population constants, some of the Group B simulation errors may reflect errors in these population values. In particular, *I_B _*can have a very wide range of patient-specific values, and may also vary over time and patient condition [37]. However, if this difference from the assumed value was significant, the variability in Figure 4 would have been potentially greater than observed. Similarly, the value of *k_I _*(endogenous insulin suppression by exogenous administration) can also vary by time and condition [37], as well as across cohorts administering different levels of insulin (Tables 4 and 5). However, while this may be one possible cause of the slight shift seen in Figure 6 for the Glucontrol B results, significant differences between model and clinical behaviour would have been evident in Figure 4 and in the prediction errors and glycaemic outcomes of Figures 5, 6 and 7.

A further and potentially more likely cause is evident in Figure 6 where the most mismatched line of the three results is for the clinical results for Glucontrol B, where the simulation results are more similar. The fact that the clinical data are lower than the simulations in this region could indicate non-compliance in timing or dosing of insulin, or simple overriding of the protocol recommendations by clinical staff. Computer simulations will always follow protocols exactly as instructed. Hence, the self validation error captures both model and compliance errors, which are clearly evident in Table 5 where insulin doses and protocol-specified measurement frequency are very different from the actual measurement case. This last point is critical because reduced measurements in the B protocol would not reduce insulin as fast as the per protocol case, resulting in lower clinical BG levels. The actual measurement self-validation simulation for Glucontrol B is much closer to the clinical data, having accounted for this effect.

For the cross validation, Protocol A on Group B is a very good match with errors similar to the self validation results for Group A. In addition, Protocol B on Group A virtual patients is within a similar range as the Group B self validation and close to the slope and trends of the clinical data. Thus, the insulin sensitivity independence assumption behind this virtual trials approach holds, independently validating this concept and the virtual trial method based on this model.

Differences between self and cross validation results are ascribed here to remaining differences between patient groups, despite clinical matching. The main notable difference pointed out in the results and Tables 4 and 5 is the difference in nutrition given each cohort. The virtual trials approach here treats each group as being treated differently, including the carbohydrate and glucose infusions administered. These infusions were patient-specific and specified based on local and individual clinician standards, rather than per a protocol of any type. Thus, they were kept for each patient. As a result, Glucontrol B patients with the higher target had 2.6× higher glucose administration, which in cross validation was offset by 3.2× more insulin in the virtual trials. Differences in insulin rates between per protocol (as the cross validation was done) and per actual measurement rates makes these differences almost equal at 2.6× higher glucose administration and 2.4× greater insulin required to achieve the almost identical glycemic outcomes. Hence, the patients display similar overall insulin sensitivity, and the virtual trials took independently treated, matched patients and achieved the same outcome despite different initial treatments in the clinical data used to create the virtual patient. More specifically, nutritional treatment differences, within reason, did not affect or influence the results outside of expectations.

More importantly, the relatively small differences show the strength of model-fitted insulin sensitivity as a description of patient metabolic state, rather than as a therapy-specific parameter value. Other causes for remaining differences may also be a function of remaining model approximations or errors. As noted, inter-patient variability in some fixed model parameters is at least one cause of model limitations and errors. However, the limited glucose data with no added or real time insulin data limits the ability to uniquely identify these parameters [27,32].

Finally, this paper shows the potential for TGC protocols to be readily optimised and implemented using model based TGC. The low prediction errors indicate an ability to minimize the risk of hypoglycaemia as well as provide tight control. Even though some TGC clinical trials have not achieved any benefit from TGC [12,38], only 2 protocols have been first optimized with virtual trials [11,17,21]. Both delivered safe, effective TGC with reduced or zero hypoglycaemia.

## Conclusions

This paper presented the analysis and validation of an in silico virtual patient and model-based virtual trials methodology. The validation approach, as presented, is readily generalized. It takes advantage of a set of independent clinical data comprised of two clinically matched cohorts treated with two different TGC protocols with two different glycemic targets. Three main conclusions can be drawn:

• Self validation indicated a clinically insignificant error in these virtual patient methods due to model and/or clinical compliance. They also showed the impact of some non-compliance independent of model error.

• Cross validation clearly showed that the virtual patient methods and models enabled by patient-specific *S_I_(t) *profiles are effective and the assumption that the *S_I_(t) *profiles are independent of the clinical inputs used to generate them holds.

• Thus, the virtual patients and in silico virtual trial methods presented are validated in their ability to accurately simulate, in advance, the clinical results of an independent TGC protocol, directly enabling rapid design and optimisation of safe and effective TGC protocols with high confidence of clinical success.

Overall, this study further shows the potential and capability of model-based, data driven in silico methods to aid protocol design, as well as the potential for models to provide accurate, safe and effective real-time TGC.

## Competing interests

The authors declare that they have no competing interests.

## Authors' contributions

JGC, FS, GS, ALC and JL conceived and developed the models and this analysis. FS, SP and ALC did most of the computational analysis with input from JGC, CGP, TD and KTM. J-CP supplied the data and Glucontrol protocol information. JGC, FS, ALC drafted the manuscript primarily although all authors made contributions. All authors approved the final manuscript.
